# ARID1B/SUB1‐activated lncRNA HOXA‐AS2 drives the malignant behaviour of hepatoblastoma through regulation of HOXA3

**DOI:** 10.1111/jcmm.16435

**Published:** 2021-03-08

**Authors:** Gongbao Liu, Baihui Liu, Xiangqi Liu, Lulu Xie, Jiajun He, Jingjing Zhang, Rui Dong, Duan Ma, Kuiran Dong, Mujie Ye

**Affiliations:** ^1^ Department of Pediatric Surgery Children’s Hospital of Fudan University Shanghai China; ^2^ Key Laboratory of Neonatal Disease Ministry of Health Shanghai China; ^3^ Department of Medical Imaging Nanjing Hospital of Chinese Medicine Affiliated to Nanjing University of Chinese Medicine Nanjing China; ^4^ Key Laboratory of Metabolism and Molecular Medicine Ministry of Education Department of Biochemistry and Molecular Biology Institute of Biomedical Sciences Collaborative Innovation Center of Genetics and Development School of Basic Medical Sciences Fudan University Shanghai China; ^5^ Shanghai Key Lab of Birth Defect Children’s Hospital of Fudan University Shanghai China

**Keywords:** chromatin remodelling factor, hepatoblastoma, lncRNA, tumorigenesis

## Abstract

It has been becoming increasingly evident that long non‐coding RNAs (lncRNAs) play important roles in various human cancers. However, the biological processes and clinical significance of most lncRNAs in hepatoblastoma (HB) remain unclear. In our previous study, genome‐wide analysis with a lncRNA microarray found that lncRNA HOXA‐AS2 was up‐regulated in HB. Stable transfected cell lines with HOXA‐AS2 knockdown or overexpression were constructed in HepG2 and Huh6 cells, respectively. Our data revealed knockdown of HOXA‐AS2 increased cell apoptosis and inhibited cell proliferation, migration and invasion in HB. Up‐regulation of HOXA‐AS2 promoted HB malignant biological behaviours. Mechanistic investigations indicated that HOXA‐AS2 was modulated by chromatin remodelling factor ARID1B and transcription co‐activator SUB1, thereby protecting HOXA3 from degradation. Therefore, HOXA‐AS2 positively regulates HOXA3, which might partly demonstrate the involvement of HOXA3 in HOXA‐AS2‐mediated HB carcinogenesis. In conclusion, HOXA‐AS2 is significantly overexpressed in HB and the ARID1B/HOXA‐AS2/HOXA3 axis plays a critical role in HB tumorigenesis and development. These results might provide a potential new target for HB diagnosis and therapy.

## 1 INTRODUCTION

Hepatoblastoma (HB), which usually originates from immature liver precursor cells, is the most common liver tumour in children, accounting for more than 65% of childhood liver malignancies and about 1% of all childhood tumours.^1^, ^2^, ^3^, ^4^ Despite the increasing diversity of treatments, the prognosis of some patients with poor classification remains unsatisfactory.^5^, ^6^, ^7^, ^8^ Hence, further understanding of the development of HB is needed to improve diagnosis, prevention and treatment. However, the etiology of HB is unclear; thus, it is necessary to explore the mechanisms underlying the pathogenesis of HB.

Long non‐coding RNAs (lncRNAs) are generally defined as RNA transcripts longer than 200 nt that do not code for proteins but are ubiquitously expressed in mammalian genomes and participate in a variety of biological processes, such as chromatin remodelling and transcription regulation, among others.^9^, ^10^ It is reported that some lncRNAs are closely linked to a variety of tumour‐associated biological processes, such as tumour initiation and progression.^11^, ^12^, ^13^ Given the importance of lncRNAs in cancer, in the current study, we focused on lncRNA HOXA‐AS2, which was highly expressed in our previous research by lncRNA microarray analysis. HOXA‐AS2 is 1048 nt long and locates on chromosome 7 situates between HOXA3 and HOXA4 in the HOXA cluster. HOXA‐AS2, which mainly promotes cell proliferation, but also interacts with the enhancer of zeste homolog 2 of polycomb repressive complex to repress gene expression, is previously shown to promote the development of cancers of the liver and stomach.^14^, ^15^, ^16^, ^17^ However, the biological functions of HOXA‐AS2 in HB have not yet been reported. In our previous report, lncRNA screening shows that HOXA‐AS2 is up‐regulated in HB, and in vitro experiments have shown that HOXA‐AS2 is associated with multiple malignant biological behaviours of proliferation, apoptosis and invasion.^15^, ^18^ The role of HOXA‐AS2 may be involved in the stability of HOXA3 or the function of other oncogene products that bind to it.^17^ These findings indicate that HOXA‐AS2 is an oncogene in HB tumorigenesis and may be a potential biomarker for HB diagnosis and therapy.

## 2 MATERIALS AND METHODS

### 2.1 Human tissues and HB cell lines

Human HB and adjacent non‐tumour liver tissues were collected from patients treated at the Children's Hospital of Fudan University. The study protocol was approved by the Institute Research Ethics Committee of the Children's Hospital of Fudan University (Shanghai, China), and informed consent was acquired from each patient. Tumour tissues were pathologically confirmed as HB. HepG2 and Huh6 cells were obtained from the Cell Bank of the Chinese Academy of Science (Shanghai, China) and cultured in Dulbecco's modified Eagle's medium (DMEM) supplemented with 10% foetal bovine serum (FBS) under a humidified atmosphere of 5% CO_2_/95% air at 37°C. All cell culture dishes and plates were purchased from Xinyou Biotechnology (Hangzhou, China).

### 2.2 Quantitative real‐time polymerase chain reaction (qRT‐PCR)

Total RNA was extracted with TRIzol reagent (Takara Bio, Inc, Shiga, Japan), and 1 μg was used to synthesize complementary DNA (cDNA) in a 20‐μL system using the cDNA Reverse Transcription Kit (Takara). The cDNA was diluted by fivefold and then subjected to qRT‐PCR analysis, which was performed with SYBR Green PCR Master Mix (Takara Bio, Inc) and the StepOne Real‐Time PCR System (Thermo Fisher Scientific, Waltham, MA, USA) with the following parameters: an initial denaturation step at 95°C for 10 minutes, followed by 38 cycles at 95°C for 60 s and 60°C for 15 s. Glyceraldehyde 3‐phosphate dehydrogenase was used as an internal control. The following primer pairs were used for qRT‐PCR analysis: HOXA‐AS2, forward (F), 5′‐GCT TTC TGG GAG TGG GAG AT‐3′, and reverse (R), 5′‐CTG AGT GAA GGG GTA GTC GG‐3′; HOXA3, (F) 5′‐GCC AAT CTG CTG AAC CTC AC‐3′ and (R) AGA GTT CAG ATA GCC ACC GG; GAPDH, (F) 5′‐GGA GCG AGA TCC CTC CAA AAT‐3′ and (R) 5′‐GGC TGT TGT CAT ACT TCT CAT GG‐3′; ARID1B, (F) 5′‐GGC CGT CCC GGA GTT TAA TAA‐3′ and (R) 5′‐CGG AGT GCA TCA TCC CCA T‐3′; and SUB1, (F) 5′‐TCA AGC TCT TCT GGC AGT GA‐3′ and (R) 5′‐GAA GAT GAC AGG GCT CTC GA‐3′. Data were analysed using GraphPad Prism 5 software (GraphPad Software, Inc, La Jolla, CA, USA). Relative gene expression was determined by the 2^−△△Ct^ method.

### 2.3 Construction of stably transfected cell lines

HOXA‐AS2 shRNA plasmids were designed and constructed by Genomiditech (Shanghai, China) with the pGMLV‐SC5 RNAi vector. The shRNA targets for HOXA‐AS2 are as followed: sh1,5′‐GCGCTCTGCAGACAAATAAAC‐3′; sh2,5′‐GGACACGTTTCTATGCCTTAC‐3′; sh3,5′‐GCCTTCCTACTCTTTGGAACT‐3′. HOXA‐AS2 and HOXA3 overexpression was conducted using the PCDH vector. Lentivirus packaging was performed in 293T cells with Lipo293F™ Transfection Reagent (Beyotime Institute of Biotechnology, Haimen, China). Following viral infection and puromycin screening, stable HepG2 and Huh6 cells were acquired.

### 2.4 Cell apoptosis detection

Cells were digested with trypsin without EDTA; then, it was collected and washed twice with pre‐cooled PBS. Suitable cells were transferred into 5‐mL flow tubes, and 5 μL annexin V‐PE and 5 μL 7‐AAD (Cat. No 40310ES20, YEASEN, Shanghai, China) were added and mixed gently. Tubes were protected from light at room temperature for 20 minutes. Samples were tested by flow cytometry within 1 hour.

### 2.5 Cell proliferation and viability assay

Cell proliferation was detected using the CCK‐8 kit (Shanghai Yeasen Biotechnology Co., Ltd., Shanghai, China) in accordance with the manufacturer's instructions. Briefly, cells were cultured in a 96‐well plate at a concentration of 1000 cells in 100 µL of medium. The Cell‐Light™ EdU Cell Proliferation Detection Kit (RiboBio Co., Guangzhou, China) was also used to detect cell proliferation. Cells were cultured in 24‐well plates for 2 hour at an EdU (5‐ethynyl‐2′‐deoxyuridine) concentration of 50 µmol/L. Then, the cells were fixed with 4% formaldehyde for 30 minutes at room temperature and permeabilized with 1.0% Triton X‐100 (Thermo Fisher Scientific) for 15 minutes. Afterwards, the cells were stained with 100 µL of Hoechst33342 Fluorescent Stain (Thermo Fisher Scientific) for 25 minutes and visualized under a fluorescent microscope.

### 2.6 Cell migration and invasion assay

The cell migration and invasion assays were performed with 24‐well culture plates with 8‐μm micropore inserts. The cells were subjected to starvation treatment before the assays. For the migration assay, 1 × 10^5^ HB cells were placed into the upper wells without FBS for 48 hours. For the cell invasion experiments, the upper wells were coated with Matrigel diluted by five times DMEM (cat. no. 356234; BD Biosciences, San Jose, CA, USA); then, 2 × 10^5^ HB cells were placed into the upper wells without FBS for 48 hours. The cells that had adhered to the wells were fixed with 4% paraformaldehyde and stained with 0.5% crystal violet. Five photographs were taken by microscope (Olympus) and counted by Image J software per field of view randomly.

### 2.7 Western blot analysis

Cell proteins were extracted with radioimmunoprecipitation assay buffer (Beyotime Institute of Biotechnology). After blocking with 8% skim milk, the membranes were incubated with the primary antibodies at 4°C overnight. After washing three times with Tris‐buffered saline with Tween 20, the membranes were incubated with the relative secondary antibody (dilution, 1:5000) for 1 hours at room temperature. The signalling of proteins was detected with an Enhanced Chemiluminescent Reagent Kit (New Cell & Molecular Biotech, Suzhou, China). The antibody information was as follows: HOXA3 (Santa Cruz, sc‐374237), ENO1 (Abcam, ab155102), SFN (Abcam, ab193667), TRIM29 (Abcam, ab108627), ARID1B (CST, 92964S), SUB1 (Abcam, ab154852), GAPDH (Proteintech, 60004‐1‐Ig), Tubulin (Proteintech, 10068‐1‐AP), goat anti‐rabbit IgG (CWBIO, CW0156) and goat anti‐mouse IgG (CWBIO, CW0110).

### 2.8 CRISPR CAS9

Guide RNAs were cloned into the LentiCRISPR v2 vector (a gift from Duan Ma'lab of Fudan University, catalog number, 125836; Addgene, Inc, Watertown, MA, USA) using *Bsm*BI (catalog number R0580S; New England Biolabs, Ipswich, MA, USA) for digestion and T4 DNA ligase (catalog number M0202S; New England Biolabs) for ligation. The CRISPR‐CAS9 target sequences for HOXA3 were as follows: SG1 5′‐GGCATTATAAGCGAACCCGT‐3′ and SG2 5′‐TTCGTCCAAGCGCGCGCGCA‐3′. The following oligonucleotide pairs were used for PCR amplification of sequences containing gRNAs: SG1 F‐5′‐GCCAAGTCGTGCTGCTAAAT‐3′/R‐5′‐TTTGCATCGCGTTGTT‐TCAC‐3′ and SG2 F‐5′‐GGTGGAGCTGGAGAAAGAGT‐‐3′/R‐5′‐AGAGTTCAGATA‐ GCCACCGG‐3′.

### 2.9 Tumour xenograft experiments

HepG2 cells were expanded and cultured until the number of cells met the requirements of animal experiments. The cells were collected, washed with pre‐cooled PBS and made into a single cell suspension with pre‐cooled PBS. Then, 2 × 10^6^ cells were inoculated subcutaneously with 5‐week‐old BALB/c nude mice using an inoculation needle. Four weeks later, the mouse cervical vertebrae were dislocated and tumours were quickly removed and photographed.

### 2.10 Immunohistochemical analysis

Immunohistochemical analysis of the HB tissue samples was performed with a standard procedure. Tissue sections were incubated with primary antibodies against ARID1B (Abcam, ab57461) and HOXA3 (Abcam, ab230879) at 4°C overnight.

### 2.11 Statistical analysis

All assays were repeated three times. All results are presented as the mean ± standard deviation (SD). Groups were compared using Student's *t* test. A probability (*P*) value of < .05 was considered statistically significant. All statistical analyses were performed with Prism software (version 5.0; GraphPad Software, Inc, San Diego, CA, USA).

## 3 RESULTS

### 3.1 HOXA‐AS2 was significantly up‐regulated in HB tissues and cell lines

**FIGURE 1:**
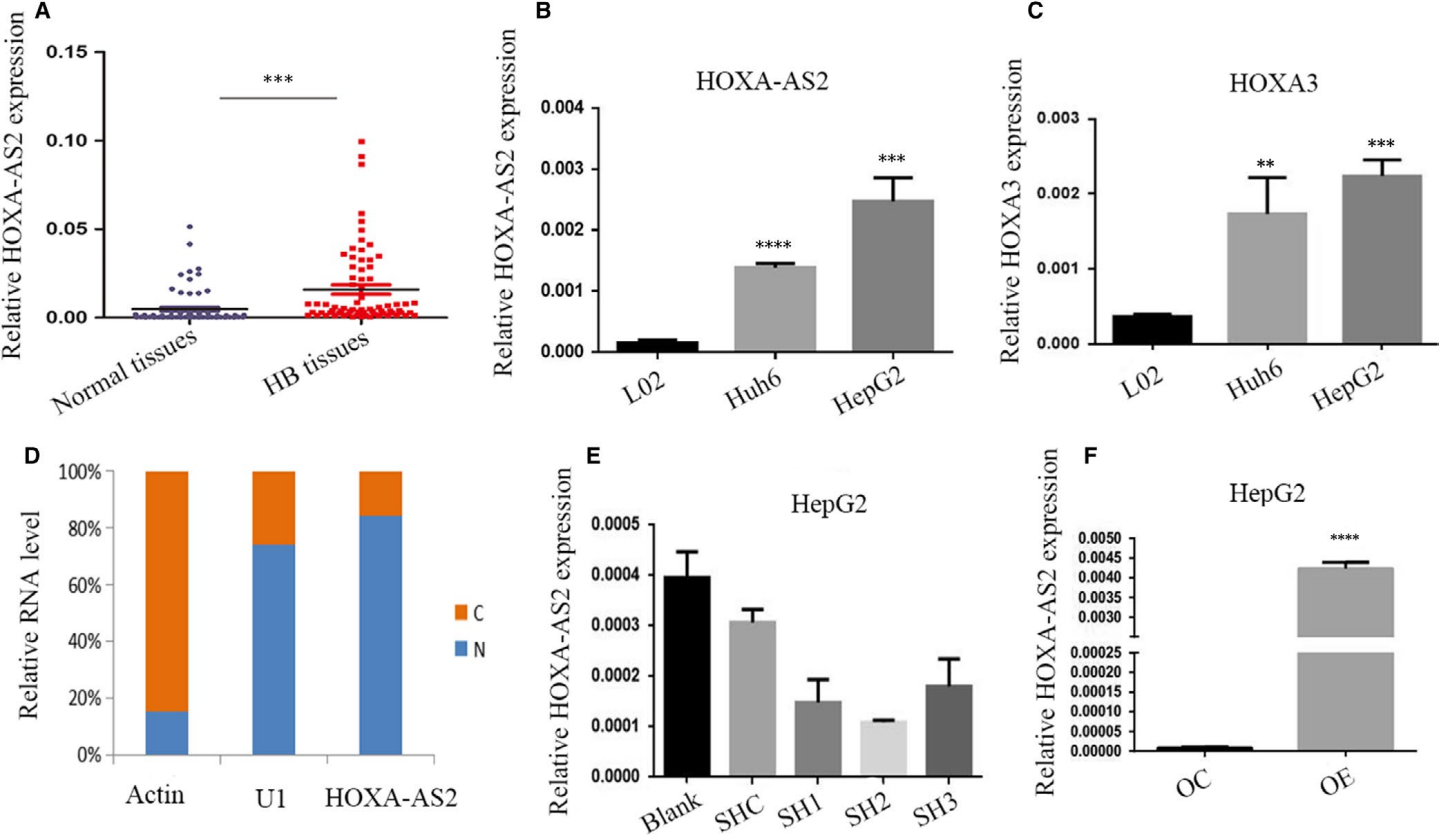
LncRNA expression and localization. A, HOXA‐AS2 expression in the children HB and normal tissues was measured by RT‐PCR and relative to that of GAPDH; B, C, Expression of HOXA‐AS2 and HOXA3 in HepG2 cell, Huh6 cells and normal liver cells L02; D, Nuclear to cytoplasmic ratio of HOXA‐AS2 variants in HepG2 cells, analysed by RT‐qPCR. U1 was analysed as a nuclear control, and actin was analysed as a cytoplasmic control, respectively; E, F, the expression of HOXA‐AS2 in HepG2 transfected with shRNA or overexpression of HOXA‐AS2 or negative control which were evaluated by RT‐PCR. Blank represents no treatment, SHC represents empty vectors for shRNA, while SH1, SH2 and SH3 represent different targets for knockdown of HOXA‐AS2, OC represents empty vector for overexpression, and OE represents overexpression of HOXA‐AS2. Data are presented as the average and SD of three independent experiments (**P < .01，***P < .001 and ****<.0001)

Our previous study found that HOXA‐AS2 was up‐regulated in HB. To determine whether HOXA‐AS2 was involved in tumorigenesis, HOXA‐AS2 expression was quantified in HB tissues. The qRT‐PCR results showed that HOXA‐AS2 expression was obviously up‐regulated in HB tissues, as compared with matched adjacent non‐tumour tissues (Figure 1A). Increased HOXA‐AS2 expression was also observed in two HB cell lines, as compared to normal L02 liver cells (Figure 1B). Meanwhile, the expression of HOXA3 was estimated via an adjacent gene of antisense lncRNA HOXA‐AS2, which found that the expression of HOXA3 was positively associated with HOXA‐AS2 levels in HB cell lines (Figure 1C). Nuclear and cytosolic separation indicated HOXA‐AS2 was mainly located in nucleus than cytosol (Figure 1D).

### 3.2 HOXA‐AS2 was an apoptosis repressor in HB

**FIGURE 2:**
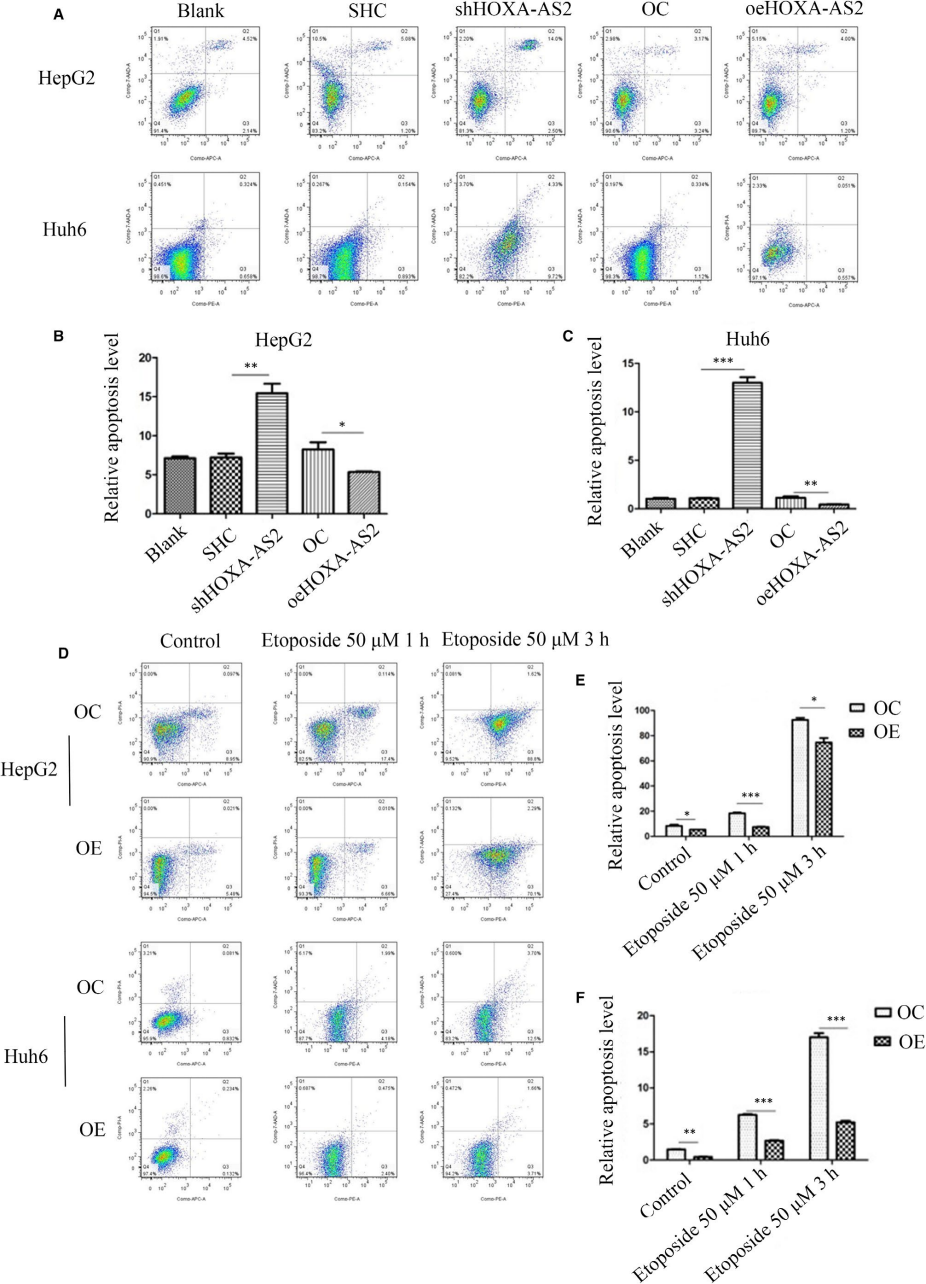
HOXA‐AS2 was an apoptosis suppressor. A, B, C Determination of the apoptosis rate of HepG2 and Huh6 cells in each group by flow cytometry; D, E, F, Flow cytometry were used to detect HepG2 and Huh6 cell apoptosis with 50 μm etoposide for 1 h or 3 h. Blank represents no treatment, SHC represents empty vector for shRNA, shRNA represents knockdown of HOXA‐AS2, OC represents empty vector for overexpression, and OE represents overexpression of HOXA‐AS2. Data shown represent the average and SD of three independent experiments. *P < .05; **P < .01; ***P < .001

To investigate the function of HOXA‐AS2, stable transfected cell lines (HepG2 and Huh6) were constructed for HOXA‐AS2 knockdown and overexpression, respectively. qRT‐PCR was used to detect the efficiency of these cell lines (Figure 1D,E). Cells were stained with the APC Annexin V Apoptosis Detection Kit with 7‐amino‐actinomycin D and analysed by flow cytometry. The percentage of apoptotic cells markedly increased in the HOXA‐AS2 knockdown cell population and slightly decreased in the population of cells overexpressing HOXA‐AS2, as compared to the scramble groups (Figure 2A‐C). To further demonstrate that HOXA‐AS2 was an inhibitor of apoptosis, etoposide was used to induce apoptosis within 1 hour or 3 hours, respectively. The results showed that the proportion of apoptotic cells was significantly reduced in the HOXA‐AS2 overexpression groups, as compared to the control groups (Figure 2D‐F).

### 3.3 HOXA‐AS2 knockdown inhibited tumour growth and colony formation

**FIGURE 3:**
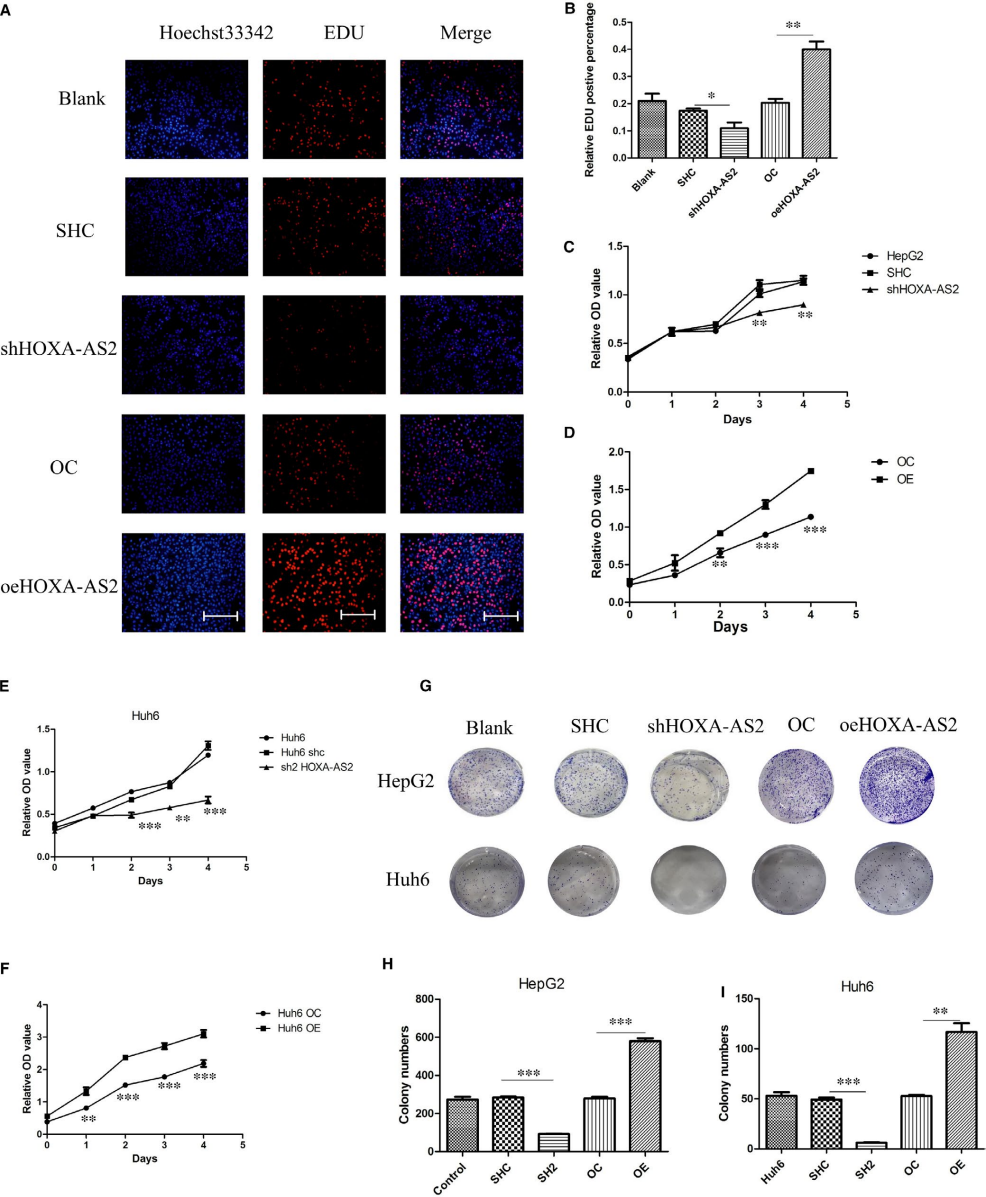
HOXA‐AS2 affected HB cell proliferation. A, B, Cell‐Light EdU DNA assay was used to reflect cell proliferation, the scale bars were 200 μm, and EdU positive represents cells that were proliferating; C, D, determination of HOXA‐AS2 silence or overexpression on the proliferation activity of HepG2 cells by CCK8 assay; E, F, determination of HOXA‐AS2 silence or overexpression on the proliferation activity of Huh6 cells by CCK8 assay; G, H, I, the colony formation of HepG2 and Huh6 cells determined by colony formation assay cell. Data shown represent the average and SD of three independent experiments. *P < .05; **P < .01; ***P < .001

The CCK‐8 assay was performed to study the effect of HOXA‐AS2 on cell proliferation. Silencing of HOXA‐AS2 markedly inhibited HB cell proliferation, as compared to the negative control group (Figure 3C,E). Moreover, overexpression of HOXA‐AS2 promoted cell proliferation (Figure 3D,F). The same phenomenon was observed with the Cell‐Light EdU DNA cell proliferation assay (Figures 3A,B and S2A). The results of colony formation analysis showed that knockdown of HOXA‐AS2 inhibited colony formation, while overexpression promoted colony formation (Figure 3G‐I).

### 3.4 HOXA‐AS2 was involved in the migration and invasion of tumour cells

**FIGURE 4:**
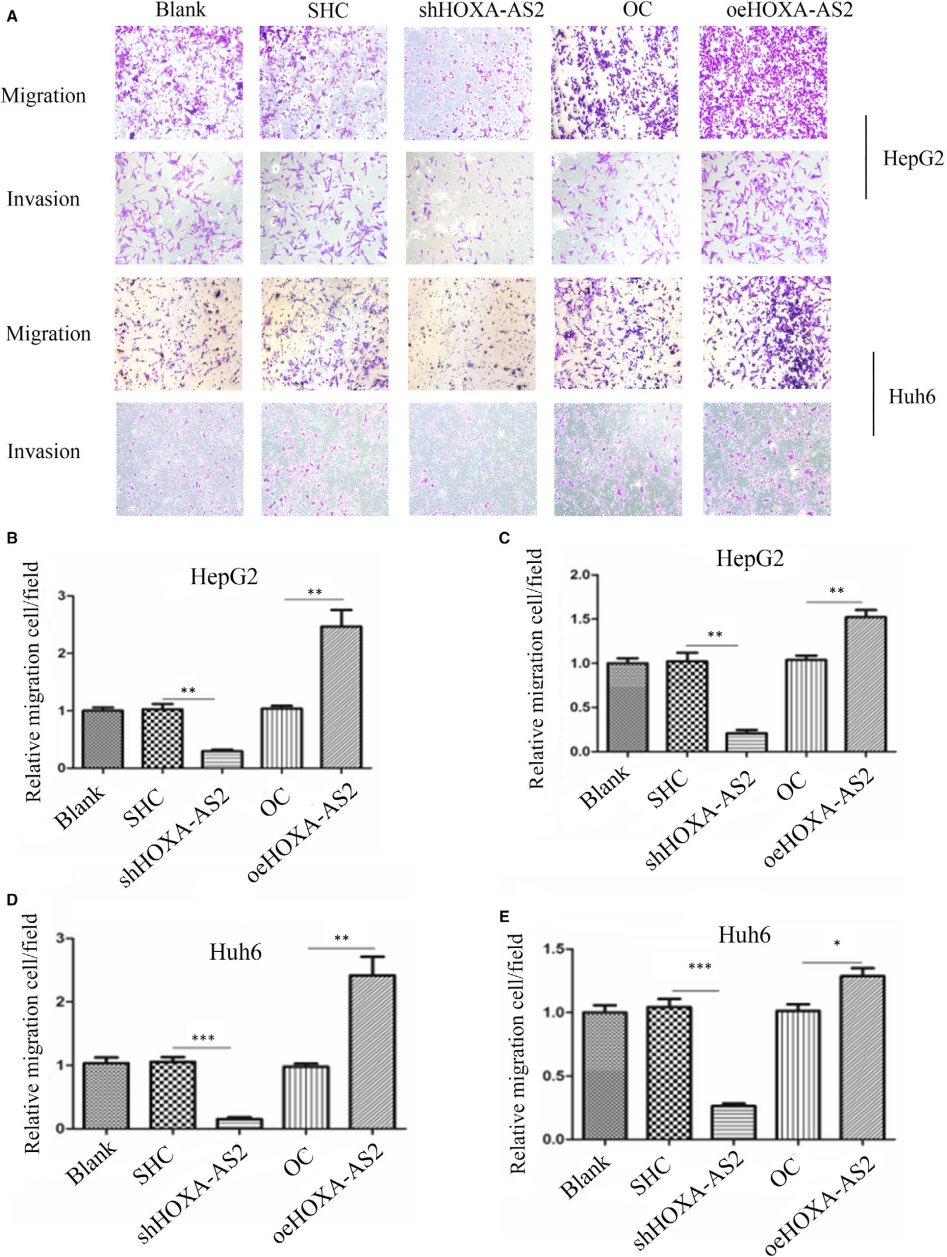
Silencing of HOXA‐AS2 expression inhibited HB cells migration and invasion. A, Transwell assays demonstrating the effect of HOXA‐AS2 down‐regulation or up‐regulation on migration and invasion in HepG2 and Huh6 cells, the microscope magnification is 100×; B, C, statistics on the relative number of migration and invasion cells in HepG2; D, E, statistics on the relative number of migration and invasion cells in Huh6. Data shown represent the average and SD of three independent experiments. *P < .05; **P < .01; ***P < .001

Transwell assays were performed to determine the effect of HOXA‐AS2 on the migration and invasion of HB cells. The results indicated that knockdown of HOXA‐AS2 inhibited HB cell migration and invasion, as compared to the control groups. However, up‐regulation of HOXA‐AS2 significantly induced cell migration and invasion (Figure 4).

### 3.5 HOXA‐AS2 up‐regulated the expression of HOXA3 by forming RNA‐RNA dimers with HOXA3 mRNA

**FIGURE 5:**
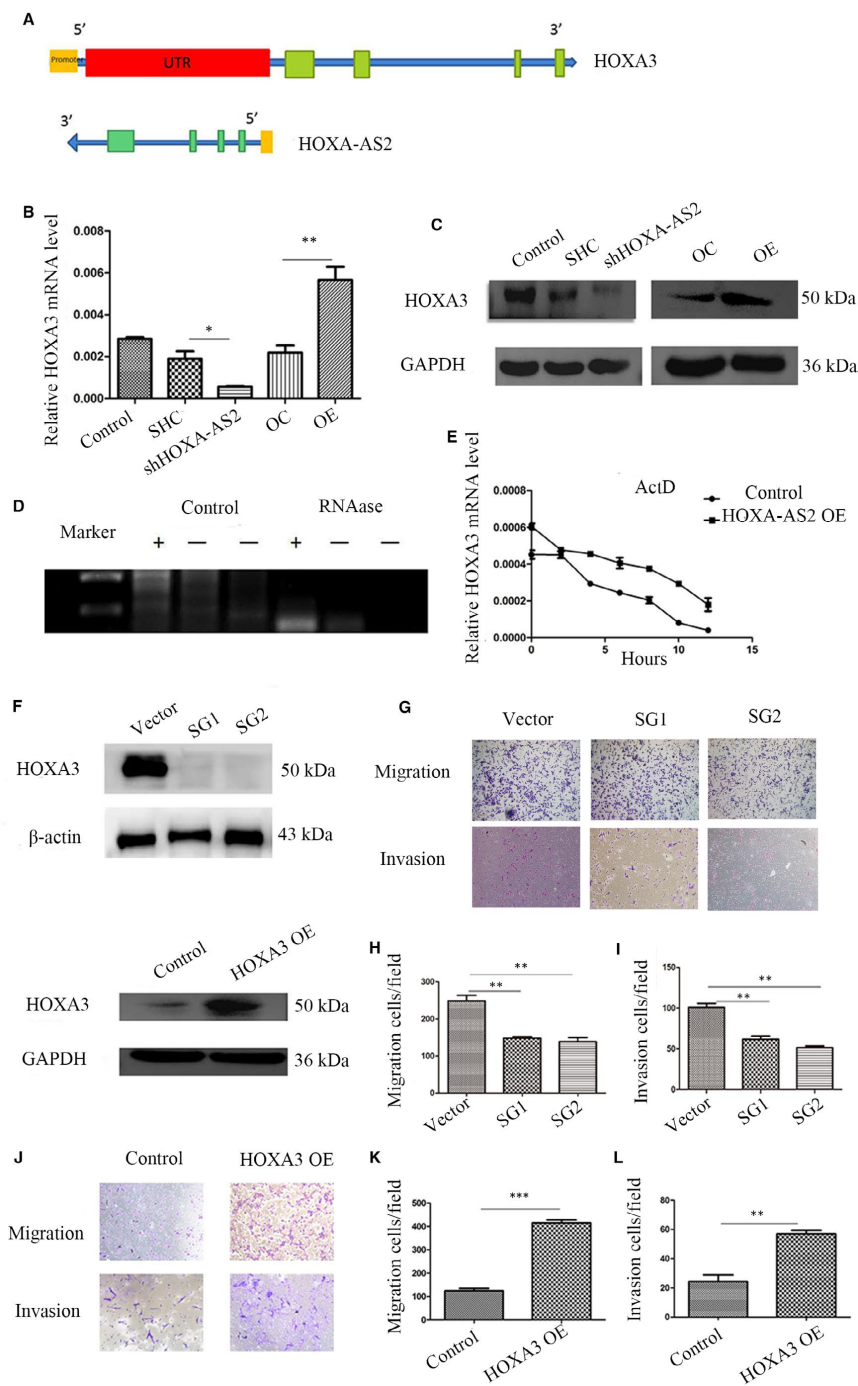
HOXA‐AS2 regulated HOXA3 expression. A, Pattern diagram showing HOXA‐AS2 and HOXA3 mRNA binding; B, qRT‐PCR for detecting HOXA3 level with HOXA‐AS2 knockdown or overexpression; C, Western blot for detecting HOXA3 level with down‐regulation or up‐regulation of HOXA‐AS2; D, ribonuclease protection assay, ‘+’ represents the region of HOXA‐AS2 binding with HOXA3 mRNA, ‘−’ represents no binding region, control group represents without RNAase, RNAase group represents adding RNAase; E, actinomycin D (1 μm) was added in HOXA‐AS2 overexpression and control HepG2 cell at 0, 2, 4, 6, 8, 10 and 12 h, respectively, in mRNA stability test; F, Western blot for detecting HOXA3 knockdown or overexpression efficiency by CRISPR/CAS9; G,H,I Transwell assays demonstrating the effect of HOXA3 knockout on migration and invasion in HepG2 cells, the microscope magnification is 100×; J, K, L, migration and invasion capacity in HepG2 cells with HOXA3 overexpression, the microscope magnification is 100×. Data shown represent the average and SD of three independent experiments. *P < .05; **P < .01; ***P < .001

HOXA‐AS2 has a 94‐bp reverse complementation region with the 5′ untranslated region of HOXA3, which may form RNA‐RNA dimers and increase the stability of HOXA3 (Figure 5A). The results of qRT‐PCR and western blot showed that knockdown of HOXA‐AS2 decreased HOXA3 expression, while overexpression of HOXA‐AS2 increased HOXA3 expression (Figure 5B,C). The results of a ribonuclease protection assay indicated that RNA‐RNA dimers inhibited the degradation of HOXA3 mRNA via RNase (Figure 5D). The mRNA stability test indicated that overexpression of HOXA‐AS2 reduced the degradation of HOXA3 (Figure 5E).

### 3.6 HOXA3 was associated with tumour migration and invasion, but not proliferation

To further investigate the function of HOXA3 in HB, stable transfected HepG2 cell lines were constructed by HOXA3 knockdown and overexpression, respectively. Western blot analysis was used to detect the efficiency of the constructed cell lines (Figure 5F). The results of the CCK‐8 assay, clonal formation experiments and EdU assay showed no significant difference among the HOXA3 knockdown groups, HOXA3 overexpression groups and relative control groups (Figure S2B‐E). However, knockdown of HOXA3 significantly inhibited HB cell migration and invasion, as compared to the control groups (Figure 5G‐I), while up‐regulation of HOXA3 markedly induced cell migration and invasion (Figure 5J‐L).

### 3.7 SFN, TRIM29 and ENO1 interacted with HOXA‐AS2 and HOXA3

**FIGURE 6:**
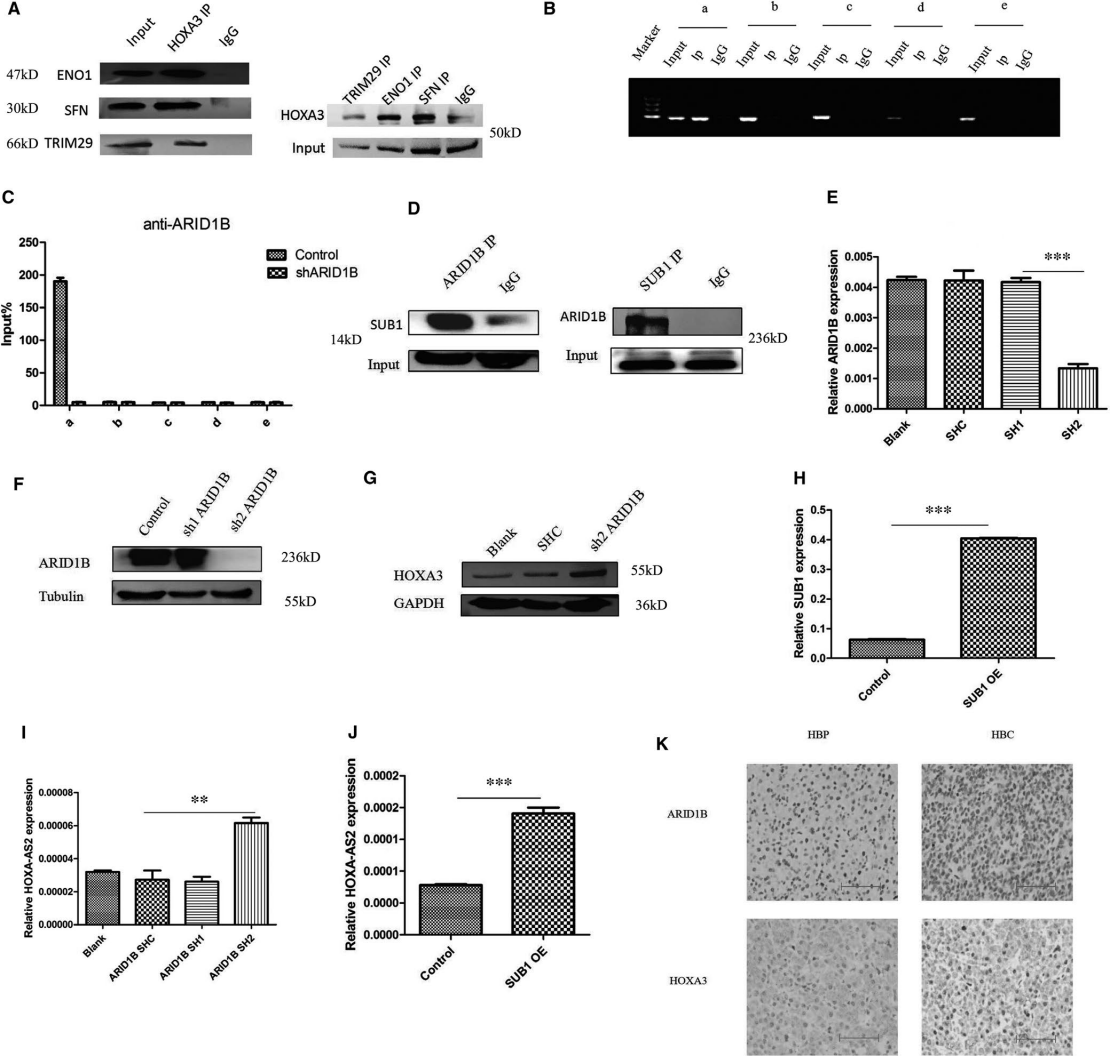
ARID1B and SUB1 co‐regulated HOXA‐AS2 expression. A, CO‐IP for HOXA3 and ENO1, SFN, TRIM29; B, C, CHIP‐PCR/QPCR for ARID1B and HOXA‐AS2 promoter(upstream of the transcription start site 2000 bp), a 1‐400 bp, b 400‐800 bp, c 800‐1200 bp, d 1200‐1600 bp, e 1600‐2000 bp, D, CO‐IP for ARID1B and SUB1, E, F, RT‐PCR and Western blot for detecting efficiency by down‐regulation of ARID1B; G, Western blot for HOXA3 with down‐regulation of ARID1B; H, J, RT‐PCR showing SUB1 overexpression efficiency and accompanying expression of HOXA‐AS2; I, RT‐PCR for HOXA‐AS2 with down‐regulation of ARID1B; K, immunohistochemistry for ARID1B and HOXA3 in HB tissues and adjacent normal liver tissues, the scale bars were 200 μm. Data shown represent the average and SD of three independent experiments. *P < .05; **P < .01; ***P < .001

To study the mechanism of HOXA‐AS2 and HOXA3 in HB, an RNA pull‐down assay for HOXA‐AS2 (Figure S1B) and immunoprecipitation of HOXA3 (Figure S1C, Figure 6A) were performed. Some gene products related to liver tumours, such as SFN, TRIM29 and ENO1, were found to bind to HOXA‐AS2 and HOXA3.

### 3.8 ARID1B and SUB1 co‐regulated the transcription of HOXA‐AS2

With the use of the University of California, Santa Cruz Genome Browser database, we found that ARID1B may bind to the HOXA‐AS2 promoter region. Chromatin immunoprecipitation coupled with qRT‐PCR confirmed that ARID1B combined with the HOXA‐AS2 promoter region at a position located 1‐400 bp upstream of the transcription start site (Figure 6B,C). Immunoprecipitation of ARID1B identified SUB1, a transcriptional co‐factor (Figure 6D). To explore the influence of ARID1B and SUB1 on HOXA‐AS2, shRNA and overexpression assays were performed, respectively (Figure 6E‐H). The qRT‐PCR results demonstrated that knockdown of ARID1B or overexpression of SUB1 induced the expression of HOXA‐AS2 (Figure 6I,J). Consistent with these findings, immunohistochemistry results found that ARID1B was down‐regulated in HB, while HOXA3 was up‐regulated, as compared with adjacent normal liver tissues (Figure 6K).

### 3.9 Knocking down HOXA‐AS2 inhibited tumour growth in vivo

**FIGURE 7:**
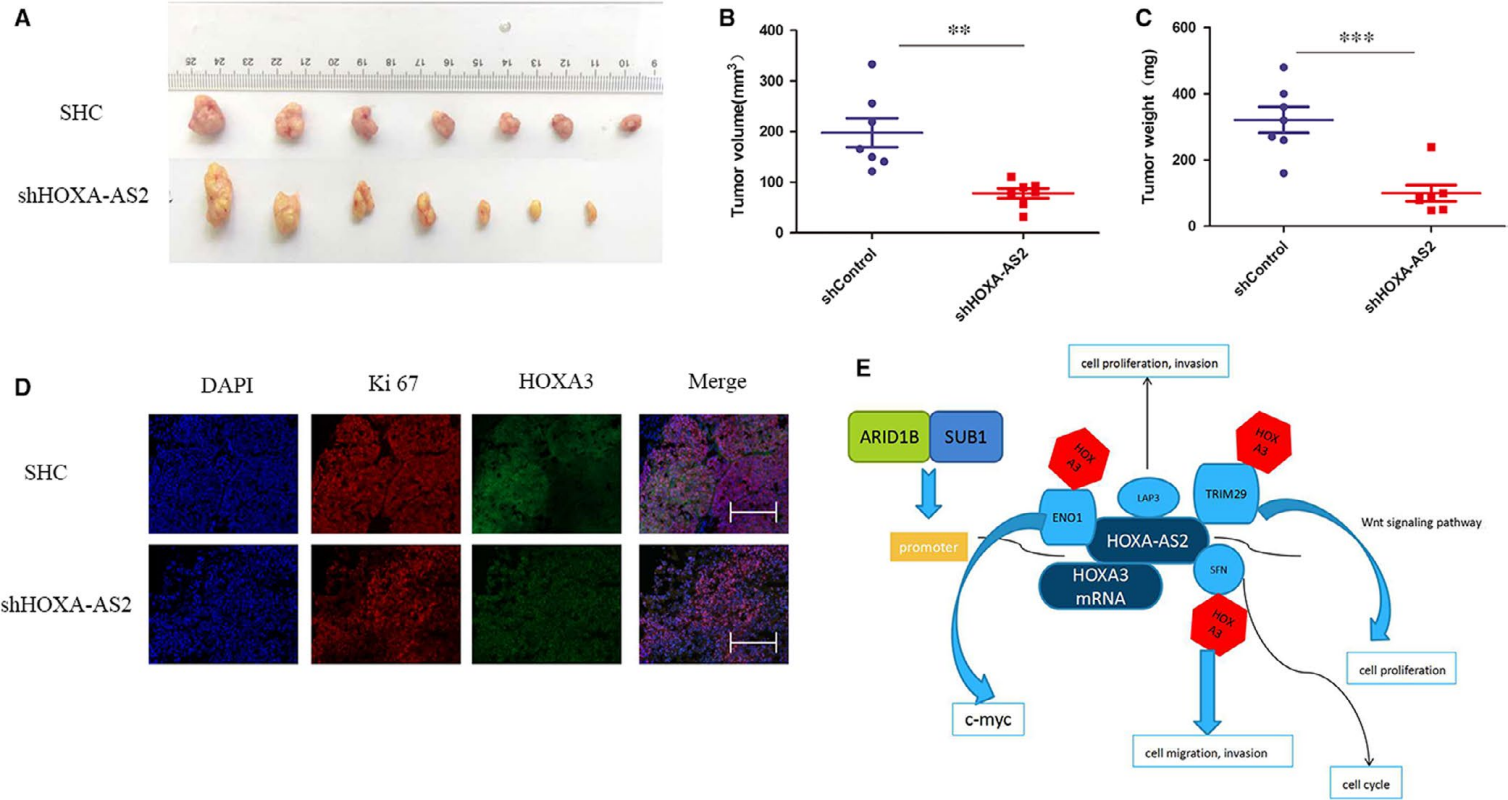
HOXA‐AS2 regulated HB cell proliferation in vivo. A, The down‐regulation of HOXA‐AS2 inhibited the tumorigenesis ability of HB cells in mice, and nude mice were randomly divided into down‐regulation of HOXA‐AS2 group and control group (n = 7); B, the tumour volume of the nude mice in HOXA‐AS2 down‐regulation group and control group; C, the tumour weight of the nude mice in HOXA‐AS2 down‐regulation group and control group; D, immunofluorescence for Ki67 and HOXA3, and the scale bars were 200 μm; E, molecular mechanism diagram we envision. **P < .01; ***P < .001

The tumour formation of nude mice showed that the tumour volume and weight were significantly lower than that of the control group after knocking down HOXA‐AS2 (Figure 7A‐C). Immunofluorescence experiments on tumour tissues formed in nude mice showed that Ki67, a marker for tumour proliferation, and HOXA3 were significantly decreased in the HOXA‐AS2 knockdown group than in the control group (Figure 7D). Based on all our research results, a mechanistic diagram centred on HOXA‐AS2 was created (Figure 7E).

## 4 DISCUSSION

The results of the present study demonstrated that up‐regulation of HOXA‐AS2 had greatly contributed to cell growth, migration and invasion in HB. Also, HOXA‐AS2 was highly expressed in HB tissues and HB cell lines, as compared to normal liver tissues and cell lines. Moreover, knockdown of HOXA‐AS2 increased cell apoptosis and inhibited cell proliferation, migration and invasion in HB. Specifically, up‐regulation of HOXA‐AS2 was likely regulated by the chromatin remodelling factor ARID1B and the transcription co‐activator SUB1. Furthermore, HOXA‐AS2 was found to bind with HOXA3 mRNA to promote HOXA3 stability. Hence, HOXA‐AS2 may be considered as a potential marker for the diagnosis and treatment of HB.

Increasing evidence has confirmed that lncRNAs play important roles in various human diseases and biological processes, especially carcinogenesis.^19^, ^20^, ^21^, ^22^ Many studies have demonstrated differential lncRNA expression in HB.^23^, ^24^, ^25^ For example, LncRNA‐TUG1 constitutes a regulatory network with miR‐34a‐5p and VEGFA that participates in the regulation of HB cell function, tumour progression and tumour angiogenesis.^8^ Wang et al found that STAT3‐activated lncRNA‐LUCAT1 drives HB cell proliferation, migration and invasion, while the reverse epithelial‐mesenchymal transition (EMT) phenotype into the mesenchymal‐epithelial transition phenotype occurs through the miR‐301b/STAT3 axis.^26^ HOXA‐AS2 was first reported as an apoptosis repressor in all transretinoic acid‐treated NB4 promyelocytic leukaemia cells.^27^ Tong et al indicated that up‐regulation of HOXA‐AS2 significantly induced tumour cell migration and invasion by affecting the EMT in colorectal cancer.^28^, ^29^ Zhao et al demonstrated HOXA‐AS2 could up‐regulate HOXA3, thereby activating the EGFR/Ras/Raf/MEK/ERK signalling pathway and decreased glucocorticoid sensitivity in acute lymphoblastic leukaemia.^17^ However, the function of HOXA‐AS2 in HB remains unclear. Actually, in our previous study, genome‐wide analysis of lncRNA expression showed that HOXA‐AS2 was significantly up‐regulated in HB tissues.

LncRNAs function as tumour suppressors or oncogenes in various human cancers, usually by regulating nearby or target genes.^30^, ^31^, ^32^, ^33^, ^34^, ^35^ In the present study, HOXA‐AS2 bound with HOXA3 mRNA and formed a dimer structure, thereby protecting the stability of HOXA3. HOXA‐AS2 has a positive regulatory effect on HOXA3, which could partly explain the involvement of HOXA3 in HOXA‐AS2‐mediated tumorigenesis. HOXA3 has been reported to participate in papillary thyroid cancer, lung adenocarcinoma, and colon cancer carcinogenesis and development. Moreover, the results of the present study showed that HOXA3 might interact with TRIM29, ENO1 and SFN, which was reportedly associated with the occurrence of liver cancer. The results of RNA pull‐down assay showed that HOXA‐AS2 combined with TRIM29, ENO1, SFN and LAP3, which was consistent with previous HOXA3 immunoprecipitation results. TRIM29 has been reported preventing hepatocellular carcinoma (HCC) progression by inhibiting Wnt/β‐catenin signalling pathway.^36^ ENO1 was correlated positively with HCC tumour size and venous invasion^37^ and could regulate c‐myc promoter activity.^38^ SFN played an important role in cancer cell resistance to chemo/radiation therapy by regulating DNA repair and cell cycle via PARP1 and CHK2.^39^ Expression of LAP3 was associated with prognosis and malignant development of human HCC.^40^ Therefore, we supposed the tumorigenic effect of HOXA3 may be achieved by binding with TRIM29, ENO1, SFN and LAP3. However, knockdown or overexpression HOXA3 could affect cell migration and invasion, but not cell proliferation. We assumed that increased cell proliferation induced by HOXA‐AS2 might be caused by the TRIM29, ENO1, SFN and LAP3 proteins or other signalling pathways, which will be the direction of our subsequent research.

## 5 CONCLUSION

In conclusion, this study elucidated the function of HOXA‐AS2 in HB. ARID1B/SUB1 activated HOXA‐AS2 expression and facilitated HOXA3 stability via interactions with TRIM29, ENO1 and SFN. These results provide references to reveal potential molecular mechanisms of HB for the future development of new diagnostic and therapeutic targets. However, other functions of HOXA‐AS2 in HB must be further elucidated.

## CONFLICT OF INTEREST

The authors declare that they have no conflict of interest.

Consent for publication: All the authors give their consent for publication.

## AUTHOR CONTRIBUTIONS

**Mujie Ye:** Data curation (lead); Formal analysis (lead); Methodology (equal); Project administration (lead); Supervision (lead). **Gongbao Liu:** Data curation (lead); Formal analysis (lead); Investigation (lead); Methodology (lead); Writing‐original draft (lead); Writing‐review and editing (lead). **Baihui Liu:** Validation (equal); Writing‐original draft (equal). **Xiangqi Liu:** Resources (supporting); Writing‐original draft (supporting); Writing‐review and editing (supporting). **Lulu Xie:** Resources (supporting); Software (supporting); Visualization (supporting). **Jiajun He:** Resources (supporting); Writing‐original draft (supporting). **Jingjing Zhang:** Visualization (supporting); Writing‐original draft (supporting); Writing‐review and editing (supporting). **Rui Dong:** Conceptualization (supporting); Supervision (supporting). **Duan Ma:** Project administration (equal); Supervision (equal); Validation (equal); Visualization (equal). **Kuiran Dong:** Funding acquisition (lead); Project administration (lead); Resources (equal); Software (equal); Supervision (equal).

## ETHICS STATEMENT

The studies involving human tissues and animal study were reviewed and approved by The Ethics Committee of Childrens' Hospital of Fudan University.

## Supporting information

Fig S1

Fig S2

FigCaption

## Data Availability

The data that support the findings of this study are available from the corresponding author upon reasonable request. Availability of supporting data: All data generated in this study are included in the manuscript.
