# Supplementary figures and images for: ARID1B/SUB1‐activated lncRNA HOXA‐AS2 drives the malignant behaviour of hepatoblastoma through regulation of HOXA3
Source: J Cell Mol Med. 2021 Mar 8;25(7):3524–36. doi: 10.1111/jcmm.16435 (PMC8034473; doi:10.1111/jcmm.16435)

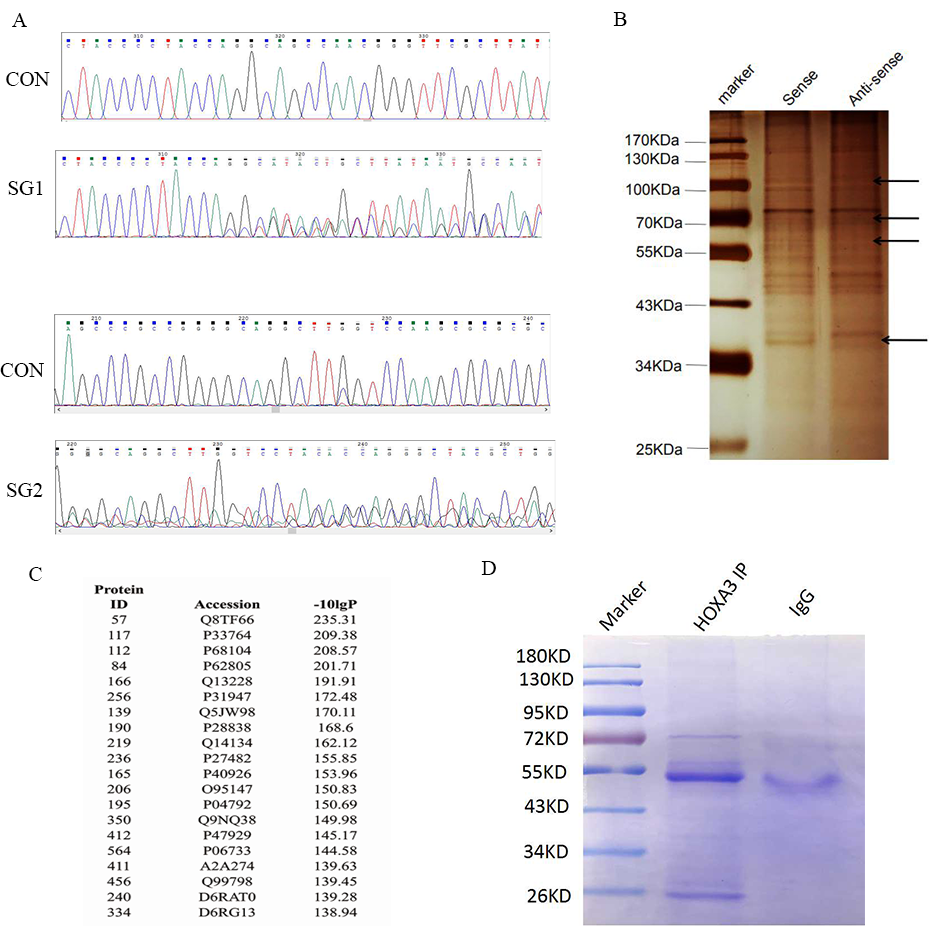

Supplement: Supplementary file 1 — Fig S1 [file JCMM-25-3524-s003.tif]

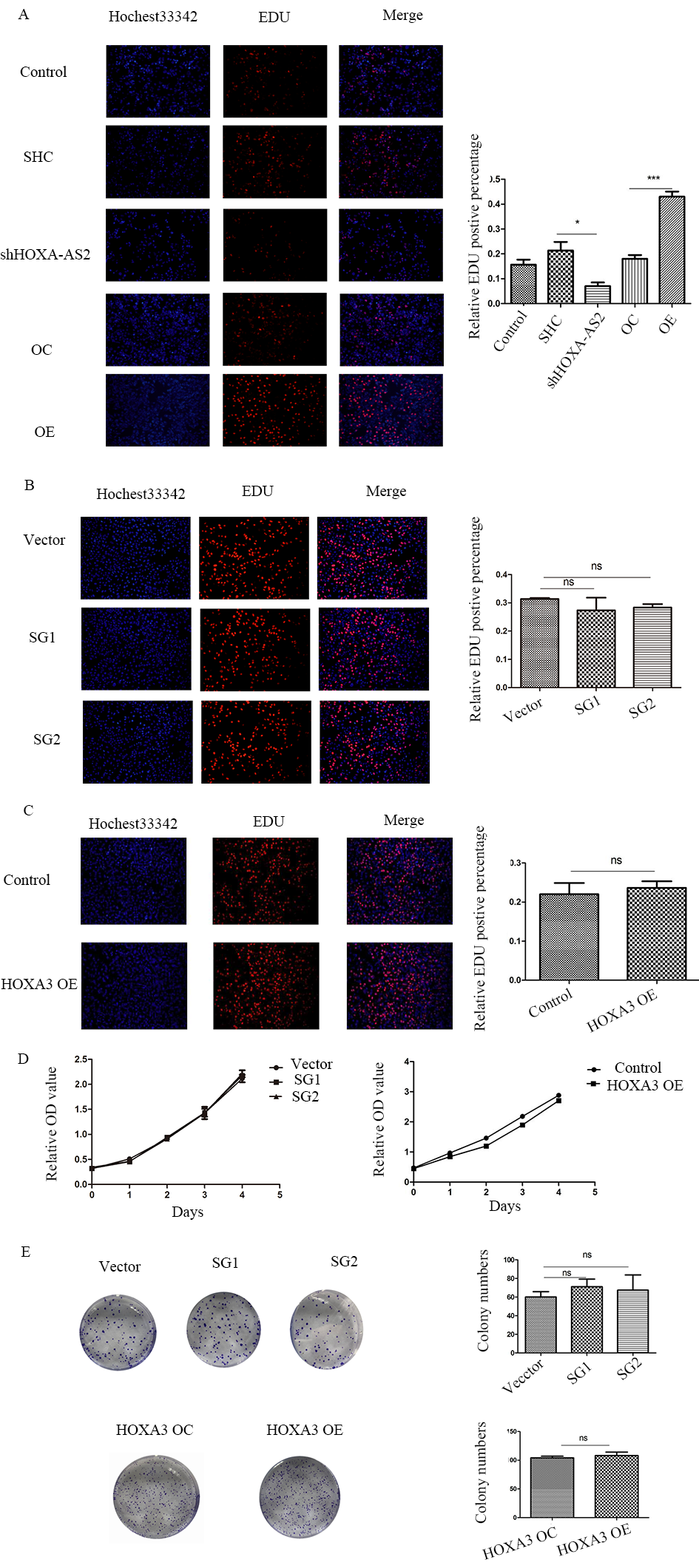

Supplement: Supplementary file 2 — Fig S2 [file JCMM-25-3524-s002.tif]
