# Supplementary material for: ARID1B/SUB1‐activated lncRNA HOXA‐AS2 drives the malignant behaviour of hepatoblastoma through regulation of HOXA3
Source: J Cell Mol Med. 2021 Mar 8;25(7):3524–36. doi: 10.1111/jcmm.16435 (PMC8034473; doi:10.1111/jcmm.16435)
Supplement: Supplementary file 3 — FigCaption [file JCMM-25-3524-s001.docx]

**Figure S1** A, DNA Sequencing results for HOXA3 CRISPR CAS9; B, RNA pull-down protein silver stain image and protein list; C, HOXA3 immunoprecipitation Coomassie blue staining

**Figure S2** A, Cell-Light EdU DNA assay for Huh6 cell with HOXA-AS2 knockdown or overexpression; B, C, Cell-Light EdU DNA assay for HepG2 cell with HOXA3 knockdown or overexpression; D, CCK-8 assay was used to measure HepG2 cell proliferation with HOXA3 down-regulation or up-regulation; E, Clone formation assay for HepG2 cell proliferation with HOXA3 knockdown or overexpression
